# A Comprehensive Understanding of *DCTPP1* as an Emerging Therapeutic Target in Liver Cancer

**DOI:** 10.3390/cimb48080770

**Published:** 2026-07-29

**Authors:** Hye In Ka, Se Ha Jang, Hyung Seok Kim, Hyun Sun Jung, Jung Woo Eun

**Affiliations:** 1 Department of Molecular Science & Technology, Ajou University, Suwon 16499, Republic of Korea; kahi22@ajou.ac.kr; 2 Department of Gastroenterology, Ajou University School of Medicine, 164 Worldcup-ro, Yeongtong-gu, Suwon 16499, Republic of Korea; csh3601@ajou.ac.kr (S.H.J.); rkawk1102@ajou.ac.kr (H.S.J.); 3 Department of Biomedical Sciences, Ajou University Graduate School of Medicine, 164 Worldcup-ro, Yeongtong-gu, Suwon 16499, Republic of Korea; 4 Department of Biochemistry, Kosin University College of Medicine, Seo-gu, Busan 49267, Republic of Korea; hyungseok.kim@yale.edu; 5 Section of Hematology, Department of Internal Medicine and Yale Cancer Center, Yale University School of Medicine, New Haven, CT 06520, USA

**Keywords:** DCTPP1, nucleotide sanitization, dNTP pool, hepatocellular carcinoma, therapeutic target

## Abstract

Balanced control of intracellular deoxyribonucleoside-triphosphate (dNTP) pools is crucial for accurate DNA replication and genome stability. Deoxycytidine triphosphate pyrophosphatase 1 (DCTPP1) is a cytosolic nucleotide-sanitizing enzyme that selectively hydrolyzes abnormal cytosine-derived triphosphates to maintain pyrimidine pool homeostasis. Dysregulated *DCTPP1* expression disrupts this balance, leading to replication stress and genomic instability. Recent studies have revealed that *DCTPP1* is frequently upregulated across various human cancers and contributes to tumor progression, stress tolerance, and chemoresistance. Particularly in hepatocellular carcinoma, an oncogenic DCTPP1/MYC feedback loop has been reported to rewire pyrimidine metabolism. This review summarizes the current understanding of DCTPP1 biology, highlights its emerging role in hepatocellular carcinoma, and discusses its potential therapeutic implications.

## 1. Introduction

Deoxyribonucleoside triphosphate (dNTP) pools are essential for accurate DNA replication and the preservation of genome integrity. Both excesses and shortages of individual dNTPs lead to elevated mutation rates, replication stress, and activation of DNA damage responses, and perturbations of dNTP balance are increasingly recognized as contributors to tumorigenesis and therapy responses [1,2].

To protect the genome from incorporation of damaged or non-canonical nucleotides, cells deploy a set of “house-cleaning” enzymes that surveil and sanitize the nucleotide pool. These enzymes include members of the Nudix hydrolase family such as MTH1 [3,4] and a separate superfamily of all-α nucleoside triphosphate (NTP) pyrophosphatases (MazG-like proteins and their eukaryotic homologs) [4,5]. They selectively hydrolyze aberrant NTP/dNTP species and thereby are thought to limit mutagenic incorporation into DNA. The concept of nucleotide “sanitization” as a distinct and evolutionarily conserved layer of genome maintenance has been articulated in structural and functional studies of these enzyme families [6]. Within the all-α NTP pyrophosphatase group, deoxycytidine triphosphate pyrophosphatase 1 (DCTPP1), also annotated as XTP3-transactivated protein A (XTP3TPA), has emerged as a cytosolic nucleotide-sanitizing enzyme with strong biochemical preference for cytosine-derived triphosphates, including modified forms such as 5-methyl-dCTP [7]. Recent structural studies and comprehensive reviews of nucleotide sanitization enzymes have placed DCTPP1 within the Nucleoside triphosphate pyrophosphohydrolase (MazG)/dCTPase-like fold and have emphasized its role in maintaining pyrimidine pool homeostasis in mammalian cells [8,9]. In addition, broader reviews highlight DCTPP1 as a component of this genome-protective enzymatic framework [4].

The trajectory of DCTPP1 research spans computational/structural annotation and subsequent cellular and biochemical characterization. Early structure-guided analyses identified RS21-C6/XTP3TPA as a MazG-like candidate with putative nucleotide-sanitizing activity; more recent biochemical and cellular studies have confirmed high selectivity of the enzyme for cytosine nucleotides and have connected DCTPP1 perturbation to altered dNTP ratios, uracil accumulation in genomic DNA, and activation of DNA damage responses in cultured cells. Collectively, these observations support the view that DCTPP1 may act as a conserved key regulator of pyrimidine pool quality whose dysregulation could influence cancer phenotypes and chemosensitivity. However, important mechanistic and tissue-specific questions remain [6].

In this review we summarize current knowledge about DCTPP1 from structural features and substrate specificity to physiological roles in maintaining replication fidelity and emerging evidence linking DCTPP1 to human malignancies. Importantly, while functional data in liver biology have been scarce [1,10], recent studies have identified an oncogenic feedback loop in liver cancer, repositioning DCTPP1 as a central player in metabolic rewiring [11]. Accordingly, this review should be interpreted as a synthesis of current evidence and a framework for hypothesis generation, rather than as definitive proof of clinical utility.

## 2. Structure and Biochemical Properties of DCTPP1

**Figure 1:**
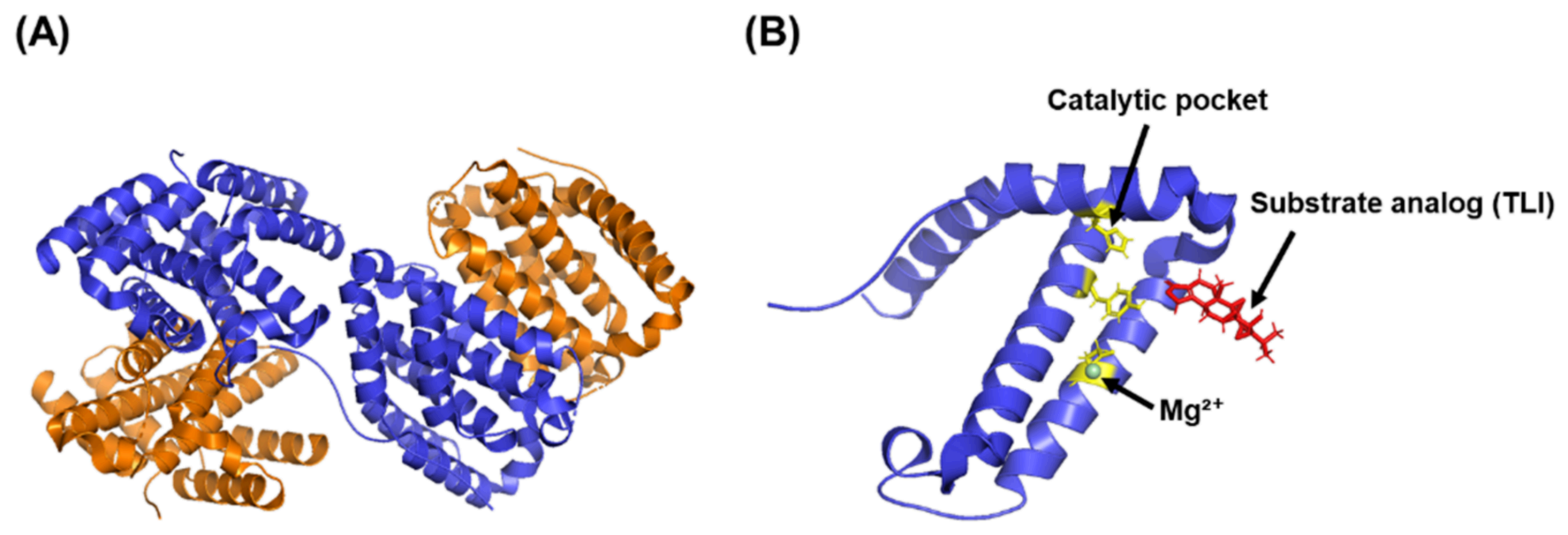
Structural organization of human DCTPP1. (**A**) Ribbon representation of the human DCTPP1 homotetramer showing its tetrameric assembly. Individual protomers are displayed in alternating colors. (**B**) Enlarged view of a representative catalytic pocket showing the bound substrate analog (TLI, red), catalytic Mg^2+^ ion (green sphere), and representative substrate-binding residues (His38, Glu63, and Tyr102; yellow). Structures were generated using PyMOL version 3.1.8 (Schrödinger, LLC, New York, NY, USA) based on the crystal structure of human DCTPP1 (PDB ID: 7MU5).

DCTPP1 is a member of the all-α NTP pyrophosphatase superfamily. Structural analyses indicate that DCTPP1 assembles as a homotetramer with a predominantly α-helical architecture, with each subunit contains a conserved MazG-like catalytic domain required for pyrophosphatase activity (Figure 1) [12]. The active site is proposed to coordinate divalent metal ions, primarily Mg^2+^, which appear to be required for efficient hydrolysis of its substrates. Structural studies of the murine homolog RS21-C6 indicate a four-helix bundle fold that forms a compact binding pocket likely involved in substrate recognition and catalysis [12]. 

Biochemically, DCTPP1 has been reported to exhibit a strong substrate preference for cytosine-derived triphosphates, including canonical dCTP such as 5-methyl-, 5-bromo-, and 5-iodo-dCTP [7]. Hydrolysis of these nucleotides to deoxycytidine monophosphate (dCMP) and pyrophosphate is thought to reduce their misincorporation into DNA and thereby preserves genome integrity [8]. Enzymatic assays have shown optimal activity at alkaline pH (pH > 7; at mildly alkaline pH (approximately 8.5–8.7)) in the presence of Mg^2+^ and markedly lower activity toward other canonical dNTPs, supporting a relatively high specificity for cytosine derivatives [7]. Structural modeling identifies His38, Glu63, and Tyr102 as key residues potentially involved in substrate recognition via hydrogen bonding and hydrophobic contacts, while the 5-methyl group of modified substrates may stabilize binding through interactions with Trp47 and Trp73 [12].

Subcellular localization studies have indicated that DCTPP1 is predominantly cytosolic but can also be detected in the nucleus and mitochondria under specific cellular conditions, including highly proliferative conditions and the S phase of the cell cycle [8,13]. This compartmental flexibility suggests a possible role for DCTPP1 in safeguarding nucleotide integrity across multiple replication-associated compartments. Crucially, recent functional evidence has expanded this paradigm to non-proliferative states, reporting that DCTPP1 plays an indispensable role in quiescent cells. DCTPP1 orchestrates dCTP pool dynamics specifically within the mitochondrial compartment, thereby safeguarding mitochondrial DNA (mtDNA) stability and preventing mutational deletion phenotypes under metabolic conditions independent of active nuclear replication [14].

At the physiological level, DCTPP1 is proposed to contribute to nucleotide-pool homeostasis by degrading abnormal cytosine nucleotides, including 5-methyl-dCTP and uracil-containing dNTPs, thereby limiting mutagenic DNA incorporation [8]. DCTPP1 loss disrupts dCTP/deoxythymidine triphosphate (dTTP)/deoxyuridine triphosphate (dUTP) balance, causing uracil accumulation, strand breaks, and replication stress that can be rescued by thymidine supplementation or dUTPase overexpression [9]. Collectively, these structural and biochemical observations establish the molecular basis for understanding the emerging role of DCTPP1 in tumor biology, which is discussed in the following sections.

## 3. DCTPP1 in Human Cancers

Accumulating evidence indicates that *DCTPP1* is aberrantly expressed in a variety of human malignancies and may be associated with tumor progression, therapeutic resistance, and patient outcomes [15]. Large-scale transcriptomic analyses from The Cancer Genome Atlas (TCGA), Genotype-Tissue Expression (GTEx), and Gene Expression Omnibus (GEO) indicate that *DCTPP1* is upregulated in many solid tumors compared with normal tissues. However, reduced expression has also been reported in several cancer types, highlighting the context-dependent nature of *DCTPP1* dysregulation.

**Figure 2:**
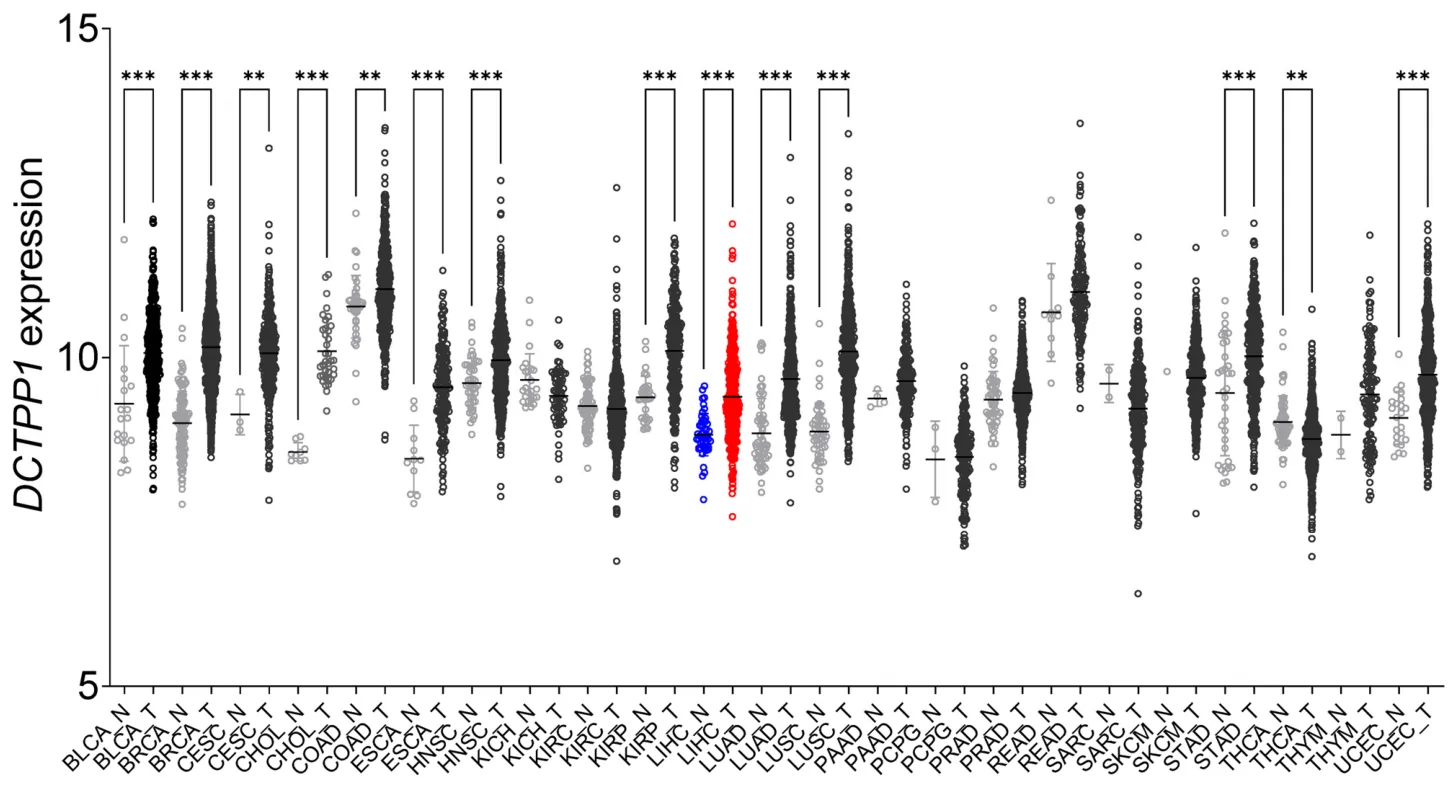
Pan-cancer expression profile of *DCTPP1*. Scatter plots illustrating *DCTPP1* expression levels (log_2_(TPM + 1)) in tumor (T, colored) and normal (N, grey) tissues across 33 cancer types. Pan-cancer gene expression data were obtained from the TCGA Pan-Cancer Atlas Hub via the UCSC Xena Browser (Illumina HiSeq RNASeqV2 dataset; *n* = 11,060). Tumor and normal samples were classified according to TCGA sample ID nomenclature (codes 01–09, tumor; 10–19, normal). Differential expression was evaluated using the Wilcoxon rank-sum test with multiple-testing correction. Data processing and statistical analyses were performed in R, and figures were generated using the ggplot2 package. LIHC is highlighted (N in blue, T in red). Statistical significance is indicated as follows: ns, not significant; ** *p* < 0.01; *** *p* < 0.001. Abbreviations for cancer types are as follows: bladder urothelial carcinoma (BLCA), breast invasive carcinoma (BRCA), cervical squamous cell carcinoma and endocervical adenocarcinoma (CESC), cholangiocarcinoma (CHOL), colon adenocarcinoma (COAD), esophageal carcinoma (ESCA), head and neck squamous cell carcinoma (HNSC), kidney chromophobe (KICH), kidney renal clear cell carcinoma (KIRC), kidney renal papillary cell carcinoma (KIRP), liver hepatocellular carcinoma (LIHC), lung adenocarcinoma (LUAD), lung squamous cell carcinoma (LUSC), pancreatic adenocarcinoma (PAAD), pheochromocytoma and paraganglioma (PCPG), prostate adenocarcinoma (PRAD), rectum adenocarcinoma (READ), sarcoma (SARC), skin cutaneous melanoma (SKCM), stomach adenocarcinoma (STAD), thyroid carcinoma (THCA), thymoma (THYM), and uterine corpus endometrial carcinoma (UCEC).

To complement the published literature, we performed a pan-cancer bioinformatic analysis using publicly available datasets from TCGA via the UCSC Xena Browser. The analysis workflow and statistical methods are described in the legend of Figure 2. Notably, elevated *DCTPP1* expression was observed in multiple tumor types. In contrast, a limited number of cancer types exhibited reduced *DCTPP1* expression in tumor tissues relative to normal controls, indicating that the direction and magnitude of *DCTPP1* dysregulation may vary in a cancer-type-dependent manner. Moreover, high *DCTPP1* expression has been consistently associated with adverse survival outcomes in several cancer types, although the underlying mechanisms appear to be context-dependent [16]. These findings are illustrated in Figure 2, and representative cancer types showing *DCTPP1* dysregulation are summarized in Table 1 [15].

In breast cancer, TCGA and GEO meta-analyses revealed that *DCTPP1* is significantly upregulated, and high expression correlates with reduced overall and recurrence-free survival [7,13,17]. In triple-negative breast cancer, FOXA1-mediated transcriptional activation of *DCTPP1* suppresses ferroptosis and promotes cisplatin resistance [18]. High *DCTPP1* expression has also been linked to an immunosuppressive tumor microenvironment by promoting M2 macrophage polarization [19]. In luminal A tumors, elevated *DCTPP1* expression has been associated with reduced chemotherapy responsiveness [20].

Similarly, in gastric cancer, *DCTPP1* overexpression has been linked to unfavorable prognosis and chemoresistance via upregulation of the multidrug resistance 1 gene (MDR1), which reduces 5-fluorouracil sensitivity [21]. Colorectal cancer data also identify *DCTPP1* as a poor-prognosis-associated factor, with natural small-molecule inhibitors reported to modulate DCTPP1-dependent metabolic reprogramming [22].

In ovarian cancer, *DCTPP1* overexpression has been associated with cisplatin resistance by reducing oxidative stress and promoting cell survival during chemotherapy [23,24]. These findings further support a role for DCTPP1 in redox homeostasis and therapy resistance across multiple cancer types. In lung adenocarcinoma, *DCTPP1* overexpression has been associated with increased tumorigenicity, chemotherapy resistance, and potential diagnostic and prognostic value [25]. Furthermore, a pan-cancer analysis underscores the context-dependent prognostic role of *DCTPP1*, with high expression associated with both favorable immunotherapeutic responses and poor outcomes in different tumor types [15]. Together, these findings indicate that DCTPP1 is a nucleotide-metabolizing enzyme with broad oncogenic relevance and suggest that nucleotide imbalance, oxidative stress tolerance, and therapy resistance contribute to malignant progression across multiple human cancers.

Recent evidence further suggests that DCTPP1 contributes to post-transcriptional regulation and stemness-related phenotypes in multiple cancer types [26]. Functional studies have shown that *DCTPP1* depletion suppresses cell proliferation, stem cell-like properties, and chemoresistance in breast and gastric cancer models [7,21]. These findings support a role for DCTPP1 in maintaining nucleotide balance and redox homeostasis while promoting tumor progression through diverse molecular mechanisms. Although these pan-cancer studies provide important biological context, the remainder of this review focuses primarily on the emerging role of DCTPP1 in hepatocellular carcinoma, where recent functional studies have begun to elucidate its biological significance.

**Table 1:** Differential Expression of *DCTPP1* in Various Cancers. Only representative cancer types with reported *DCTPP1* upregulation are included.

Cancer Type	Expression	Reference
Hepatocellular carcinoma (LIHC)	Upregulation	[11,16]
Prostate cancer	Upregulation	[27]
Ovarian cancer	Upregulation	[23,24]
Gastric cancer	Upregulation	[28,29,30]
Renal clear cell carcinoma	Upregulation	[15]
Breast cancer	Upregulation	[17,20,31,32,33]
Triple-negative breast cancer	Upregulation	[18]
Hematologic malignancy	Upregulation	[34]
Cervical cancer	Upregulation	[35]

## 4. DCTPP1 in Liver Biology and Disease

Although DCTPP1 has not been extensively studied in liver biology or hepatocarcinogenesis, several observations suggest its potential oncogenic relevance. In MYC-driven mouse models of liver tumorigenesis, *DCTPP1* expression has been reported to be elevated in MYC-overexpressing liver stem/progenitor cells, suggesting an association between *DCTPP1* expression and dedifferentiated or highly proliferative cellular states [9,36]. Consistent with these findings, DCTPP1 has been observed to accumulate in the nucleus of liver carcinoma cells, which may indicate potential nuclear roles in DNA replication or repair processes [16]. Furthermore, pan-cancer transcriptomic analyses, including hepatocellular carcinoma (HCC) datasets, indicate that *DCTPP1* expression is higher in liver tumors relative to normal hepatic tissues [15].

To further assess the clinical relevance of these observations, we performed a pan-cancer survival analysis using the publicly available Tumor Immune Estimation Resource version 3 (TIMER3) platform, and the results are summarized in Table 2. Among the analyzed cancer types, high *DCTPP1* expression was significantly associated with poor clinical outcomes in liver hepatocellular carcinoma (LIHC), ranking among the strongest prognostic associations across cancer types after multiple-testing adjustment. As these findings are derived from in silico analyses, they should be interpreted as correlative rather than indicative of causality.

Consistent with a biomarker-oriented interpretation, immunohistochemical data from the Human Protein Atlas indicate that DCTPP1 is variably expressed in human liver cancer tissues, with predominantly cytoplasmic localization and heterogeneous staining intensity across patients. This inter-patient variability highlights the potential utility of DCTPP1 as a prognostic or patient-stratification biomarker in HCC. Nevertheless, heterogeneous protein expression alone cannot establish functional dependency or therapeutic tractability, emphasizing the need for mechanistic studies to define the biological role of DCTPP1 in liver tumorigenesis.

**Figure 3:**
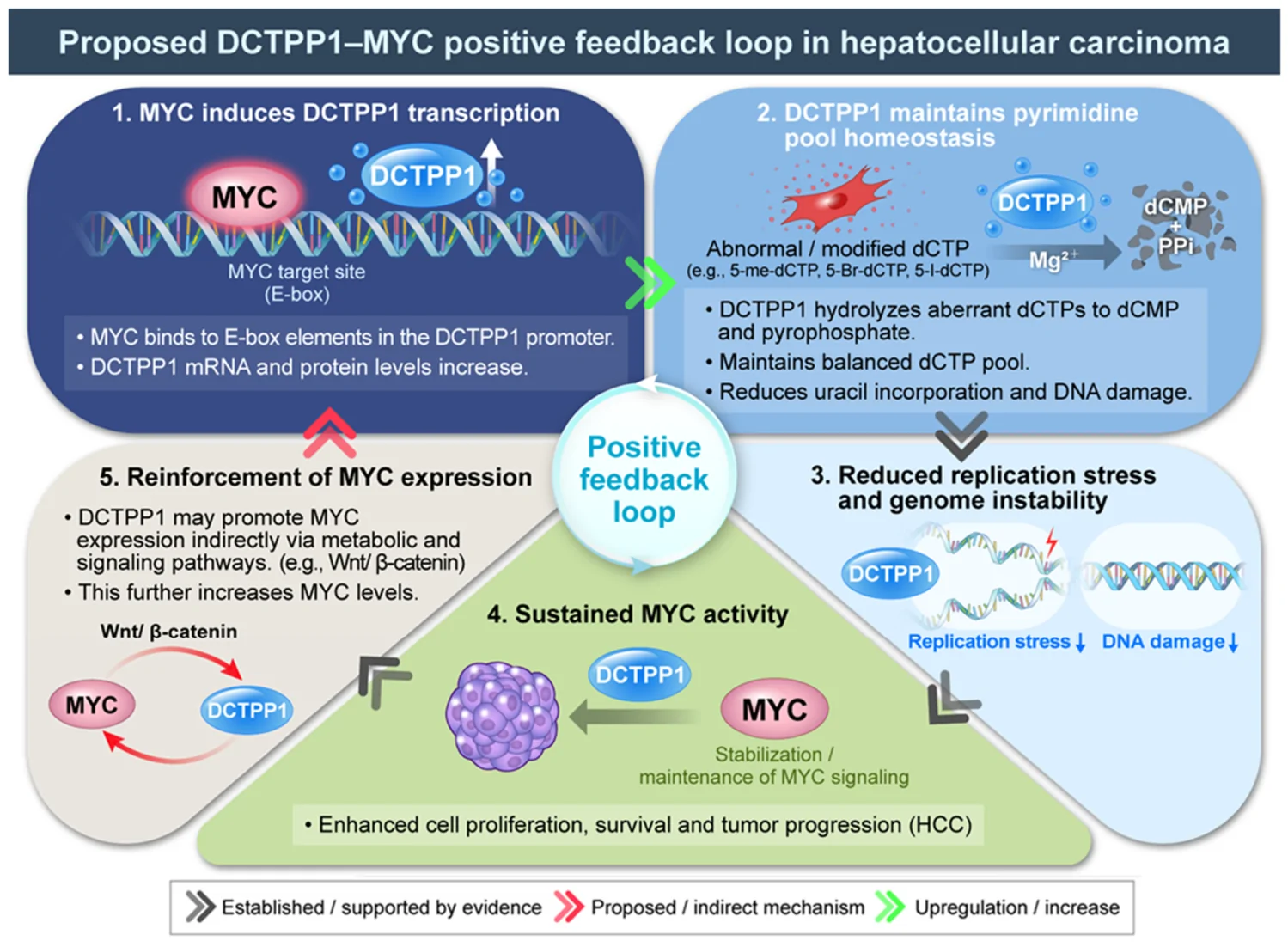
Proposed mechanistic model of the DCTPP1–MYC regulatory axis in hepatocellular carcinoma (HCC). MYC transcriptionally activates *DCTPP1* through E-box elements within the *DCTPP1* promoter, resulting in increased *DCTPP1* expression in HCC cells (Step 1). Elevated DCTPP1 maintains pyrimidine nucleotide homeostasis by hydrolyzing aberrant or modified cytosine-derived triphosphates into deoxycytidine monophosphate (dCMP) and inorganic pyrophosphate (PPi), thereby preserving deoxycytidine triphosphate (dCTP)pool balance and limiting uracil incorporation into DNA (Step 2). Maintenance of nucleotide-pool homeostasis is proposed to reduce replication stress and genome instability (Step 3), thereby supporting sustained MYC activity and promoting cell proliferation, survival, and tumor progression (Step 4). Based on recent findings, DCTPP1 may also indirectly reinforce MYC expression or signaling through metabolic remodeling and pathways such as Wnt/β-catenin, forming a proposed positive-feedback loop (Step 5). Black arrows indicate experimentally supported relationships, red arrows denote proposed or indirect mechanisms that require further experimental validation, and green arrows represent upregulation or increased activity. This schematic integrates the established biochemical functions of DCTPP1 with the current working model in HCC while highlighting hypotheses that remain to be experimentally validated. In the diagram, white upward arrows indicate transcriptional upregulation, green arrows indicate upregulation or increased activity, red curved arrows represent regulatory feedback circuits, blue downward arrows denote reduction, and grey straight arrows indicate mechanistic connections.

Despite these correlations, functional studies directly examining DCTPP1 in hepatocyte physiology or hepatocarcinogenesis remain limited. A recent study has begun to address this gap by reporting a novel oncogenic DCTPP1/MYC positive-feedback loop that may contribute to HCC progression (Figure 3).

Specifically, the study proposes a model in which MYC transcriptionally induces *DCTPP1* expression, whereas elevated DCTPP1 may maintain pyrimidine nucleotide homeostasis, thereby alleviate replication stress and support sustained MYC-driven tumor growth. Although several components remain hypothetical, this model provides a useful conceptual framework for understanding the emerging role of DCTPP1 in hepatocellular carcinoma.

At the molecular level, this regulatory axis is initiated by MYC-mediated transcriptional activation of *DCTPP1* [11]. Elevated DCTPP1 may subsequently reinforce MYC-driven metabolic adaptation by maintaining pyrimidine nucleotide homeostasis [11,37]. These findings suggest that DCTPP1 may play a broader role than nucleotide sanitization alone by integrating nucleotide quality control with oncogenic metabolic reprogramming. Beyond identifying *DCTPP1* as a downstream target of MYC, this newly identified regulatory axis provides important insights into the metabolic adaptation of hepatocellular carcinoma.

The biological significance of this regulatory axis becomes particularly evident in MYC-driven tumors, which exhibit increased nucleotide biosynthesis, accelerated DNA replication, and elevated replication stress [38,39]. These hallmarks of highly proliferative cancers create a continuous demand for balanced intracellular nucleotide pools [37,40]. Under these conditions, the accumulation of non-canonical or damaged pyrimidine nucleotides is expected to increase, thereby creating a greater dependence on nucleotide-sanitizing enzymes such as DCTPP1 to preserve replication fidelity. Within this context, DCTPP1 may function as a metabolic safeguard that preserves pyrimidine nucleotide homeostasis by preventing the incorporation of non-canonical pyrimidine nucleotides into DNA. Maintenance of nucleotide quality is proposed to alleviate MYC-driven replication stress, thereby supporting sustained tumor cell proliferation and metabolic adaptation [39,41]. Rather than acting solely as a housekeeping enzyme involved in nucleotide sanitization, DCTPP1 may therefore serve as a metabolic regulator linking nucleotide quality control with MYC-driven metabolic reprogramming in HCC [11,42]. Although this model is currently supported primarily by a single functional study, it provides a biologically plausible rationale for understanding the metabolic dependency of rapidly proliferating liver cancer cells. Collectively, these results provide functional evidence that DCTPP1 may represent a promising therapeutic candidate in hepatocellular carcinoma. Importantly, these findings extend beyond the correlative clinical observations described above but remain insufficient to establish DCTPP1 as a clinically validated therapeutic target. Additional mechanistic, pharmacological, and preclinical studies are therefore required before the therapeutic potential of DCTPP1 in hepatocellular carcinoma can be fully established.

Despite the proposed mechanistic model summarized in Figure 3, several key mechanistic questions remain unresolved [11]. Accordingly, this model should be regarded as a working hypothesis rather than a fully established signaling pathway. It remains unclear whether DCTPP1 directly regulates MYC stability or transcriptional activity, or whether the observed positive-feedback loop is mediated indirectly through metabolic remodeling. Although MYC is currently the best-characterized upstream regulator of *DCTPP1*, additional oncogenic signaling pathways may also contribute to *DCTPP1* regulation.

Moreover, MYC is a well-established downstream effector of the Wnt/β-catenin signaling pathway, one of the most frequently dysregulated oncogenic pathways in hepatocellular carcinoma [43,44,45]. Whether DCTPP1 functionally interacts with Wnt/β-catenin signaling or whether DCTPP1 dependency is preferentially associated with MYC-activated HCC remains to be determined. Addressing these questions will be essential for determining whether DCTPP1 represents a true therapeutic vulnerability rather than simply a prognostic biomarker [15]. These observations provide a basis for future studies aimed at validating the proposed DCTPP1/MYC regulatory model and determining whether DCTPP1 constitutes a genuine metabolic dependency in hepatocellular carcinoma. Considering its established biochemical role in dNTP homeostasis and replication fidelity [8], liver-focused research using conditional knockout or overexpression models is warranted. Experimental strategies may include liver- or progenitor-specific *DCTPP1* perturbation in MYC-driven HCC models, human liver organoids, or stem-cell-derived hepatocytes combined with dNTP metabolomics, uracil-incorporation assays, and replication-stress markers. Such approaches could elucidate whether DCTPP1 contributes causally to liver tumor initiation or progression and evaluate its suitability as a therapeutic target in HCC.

Importantly, although transcriptomic, immunohistochemical, and survival analyses consistently support the clinical relevance of DCTPP1 in HCC, these observations remain largely correlative. Therefore, current evidence is sufficient to support DCTPP1 as a promising prognostic biomarker and an emerging therapeutic candidate, but not yet as a fully validated therapeutic target. Future mechanistic and preclinical studies will ultimately determine whether DCTPP1 represents a genuine therapeutic vulnerability in hepatocellular carcinoma.

## 5. Targeting DCTPP1: Experimental and Therapeutic Strategies

Functional interrogation and therapeutic targeting of DCTPP1 can be approached using both genetic and pharmacological strategies. Transient small interfering RNA (siRNA) or short hairpin RNA (shRNA) mediated knockdown allows short-term phenotypic assessment and serves as an initial validation step, ideally employing multiple independent siRNAs with appropriate controls [46]. For definitive gene disruption, Clustered regularly interspaced short palindromic repeats/CRISPR-associated protein 9 (CRISPR/Cas9)-mediated knockout or allele-specific editing provides robust loss-of-function models that may be suitable for long-term and in vivo analyses [47].

Complementary to genetic approaches, small-molecule inhibitors offer the possibility of rapid and reversible modulation of DCTPP1 activity, thereby providing experimental tools to explore its potential therapeutic relevance in liver diseases and HCC. Several chemical scaffolds have been reported, including triptolide [48], Pyrcoumin [49], and synthetic series such as piperazin-1-ylpyridazines [50], and 3,6-disubstituted triazolothiadiazoles [51]. Among these, triptolide is the most well-characterized compound known to suppress DCTPP1 activity by directly binding and inhibiting its enzymatic function [48]. Importantly, triptolide’s water-soluble pro-drug Minnelide is under clinical investigation for various solid tumors including liver cancers [52,53]. Beyond these generic loss-of-function and pharmacological approaches, DCTPP1 may be of particular interest in the context of MYC-driven tumorigenesis. MYC is a master regulator of nucleotide biosynthesis, ribosome biogenesis, and DNA replication, and MYC overexpression is known to be associated with severe replication stress by imposing extreme demands on dNTP pool balance and replication fidelity [54,55]. In MYC-driven liver tumor models, elevated *DCTPP1* expression in liver stem/progenitor cell populations has been observed, raising the possibility that DCTPP1 may function as an adaptive safeguard that buffers MYC-induced metabolic and replicative stress by sanitizing damaged or excess cytosine nucleotides [36]. Under MYC-hyperactivated conditions, functional disruption of DCTPP1 would be predicted to exacerbate nucleotide imbalance, increase uracil misincorporation into genomic DNA, and amplify DNA damage signaling, thereby limiting tumor cell viability. This model positions DCTPP1 not merely as a passive metabolic marker but as a context-dependent vulnerability in MYC-driven hepatocarcinogenesis. This therapeutic rationale has now been validated empirically by the discovery of the reciprocal regulation between DCTPP1 and MYC in HCC models, where targeting the DCTPP1/MYC axis successfully disrupted pyrimidine metabolic homeostasis and suppressed tumor progression [11]. Such a hypothesis can be directly tested using combinatorial experimental systems that integrate MYC overexpression with *DCTPP1* genetic perturbation in hepatocyte or liver progenitor models, allowing discrimination between correlation and functional dependency. To ensure experimental specificity, rigorous on-target validation is essential. Standard strategies include phenocopy assays, where chemically distinct inhibitors reproduce the cellular phenotypes seen with genetic knockdown or knockout, and non-additivity or epistasis testing, where combining an inhibitor with CRISPR-mediated *DCTPP1* loss fails to produce further effects [56,57]. Rescue experiments are also critical, with re-expression of RNAi-resistant or wild-type DCTPP1 restoring the phenotype, whereas catalytically inactive mutants do not. Complementary target engagement assays such as enzyme kinetics, thermal shift assays, Cellular Thermal Shift Assay (CETSA), and chemoproteomic pulldown provide biochemical confirmation of compound binding [58,59]. Together, these orthogonal strategies—integrating genetic depletion, pharmacological inhibition, and robust validation—represent recommended experimental standards for interrogating DCTPP1 function and for assessing its potential therapeutic relevance, rather than constituting definitive proof of clinical utility. A concise summary of representative experimental strategies, chemical scaffolds, phenotypic outcomes, and validation methods is provided in Table 3.

## 6. Future Perspectives and Challenges

Biochemical and cellular studies have established DCTPP1 as an essential regulator of dNTP pool homeostasis and genome stability [8,9]. Nevertheless, critical mechanistic questions remain unanswered, particularly how DCTPP1 activity is integrated into oncogenic signaling networks, tissue-specific nucleotide metabolism, and in vivo tumor progression. In the liver, this question gains particular significance because hepatocytes display uniquely high nucleotide turnover and replication potential, driven by continuous metabolic flux and regenerative capacity. Under such regenerative conditions, tight control of nucleotide pool quality becomes especially critical, suggesting that DCTPP1 activity may be particularly important in safeguarding replication fidelity during liver regeneration and repair. Chronic hepatic injury, including viral hepatitis, steatohepatitis, and cirrhosis, creates an environment of oxidative stress, inflammation, and repeated cell proliferation that profoundly perturbs nucleotide balance and genome stability, processes closely linked to hepatocarcinogenesis [10,60,61]. In such settings, increased oxidative nucleotide damage and sustained replicative demand are expected to increase cellular reliance on nucleotide pool-sanitizing enzymes such as DCTPP1, potentially increasing the biological relevance of DCTPP1 in chronic liver disease. Although multiple cell-active and chemically distinct DCTPP1 inhibitors have been developed, including scaffolds that can enhance the cytotoxicity of nucleoside analogs, their pharmacological selectivity and translational relevance remain incompletely characterized [49,62].

Pan-cancer transcriptomic analyses and targeted knockdown studies suggest that *DCTPP1* suppression reduces proliferation and influences tumor immune and drug-response signatures across several tumor models. These observations support the hypothesis that DCTPP1 may represent a context-dependent therapeutic vulnerability rather than a universal oncogenic driver. However, given that nucleotide metabolism is profoundly reprogrammed during liver disease progression—from chronic inflammation and fibrosis to malignant transformation—it will be important to evaluate whether *DCTPP1* expression or activity correlates with hepatic disease stage or HCC subtype, particularly in the context of liver regeneration and metabolic reprogramming that accompany chronic liver injury and tumor progression. Recent multi-omic analyses in HCC cohorts have revealed that dysregulation of pyrimidine metabolism enzymes contributes to tumor proliferation and poor prognosis, suggesting that DCTPP1 could play a similar, but context-dependent, role in hepatic oncogenesis [63,64].

Importantly, the therapeutic exploitation of DCTPP1 must carefully balance antitumor efficacy with tissue-specific toxicity. Notably, systematic toxicity profiles of selective DCTPP1 inhibition in normal tissues, including the liver, remain largely undefined, underscoring the need for careful preclinical evaluation of therapeutic specificity and safety. Experience from other nucleotide metabolism targets such as thymidylate synthase and ribonucleotide reductase demonstrate that on-target toxicities, metabolic compensation, and acquired resistance frequently limit therapeutic windows [2,65,66]. These risks are particularly pronounced in the liver, where extensive metabolic redundancy, detoxification capacity, and regenerative potential may either buffer or exacerbate the consequences of DCTPP1 inhibition. Moreover, the high proliferative and regenerative capacity of hepatocytes raises the possibility that sustained DCTPP1 inhibition could impair normal hepatic repair processes or provoke adaptive metabolic rewiring, highlighting the importance of defining liver-specific toxicity profiles and safe dosing strategies early in preclinical development [67,68].

Future work should therefore prioritize the development of high-quality, selective chemical probes with validated cellular target engagement (for example, CETSA and chemoproteomic assays) and integration with orthogonal genetic models, including conditional loss- and gain-of-function systems. Rigorous functional analyses combining quantitative dNTP and uracil-incorporation assays, replication-stress markers, and DNA damage readouts will be essential to define the biological consequences of DCTPP1 modulation in physiological and oncogenic contexts. To accurately assess therapeutic specificity and toxicity, such studies should be conducted in liver-relevant experimental systems, including primary hepatocytes, hepatic organoids, and inflammation- or fibrosis-driven HCC models, which better capture tissue-specific metabolic and microenvironmental influences on DCTPP1 function.

In parallel, biomarker-guided combination strategies—such as pairing DCTPP1 inhibition with nucleoside analogs or DNA-damaging agents—may offer a rational approach to enhance therapeutic efficacy while minimizing systemic toxicity. Defining such rational therapeutic windows, together with predictive biomarkers of response and toxicity, will be critical for de-risking clinical translation and enabling the safe advancement of DCTPP1-targeted interventions into cancer therapy [2,58].

## 7. Conclusions

DCTPP1 is a conserved nucleotide-pool-sanitizing enzyme that contributes to the maintenance of replication fidelity by hydrolyzing aberrant cytosine nucleotides. Convergent structural, biochemical, and cellular studies support its role in genome maintenance and suggest that dysregulation of *DCTPP1* may be associated with tumor biology, including context-dependent effects on proliferation, stress tolerance, and drug response. However, the majority of current evidence is derived from in vitro systems, bioinformatic analyses, and non-hepatic cancer models, and therefore should be interpreted with appropriate caution. Selective chemical probes combined with orthogonal genetic approaches will be essential for establishing on-target mechanisms and validating DCTPP1 biology. At present, direct functional evidence linking DCTPP1 activity to liver physiology or hepatocarcinogenesis remains limited, particularly in the context of the biological heterogeneity and lineage plasticity of primary liver cancers [69]. Therefore, its proposed role in HCC should be regarded as a testable hypothesis rather than a fully demonstrated causal mechanism. Accordingly, DCTPP1 should not yet be considered a validated therapeutic target in liver cancer, but rather a candidate molecule whose biological and translational relevance requires further empirical validation. Future liver-focused studies using physiologically relevant experimental models and clinical specimens will be essential to determine whether DCTPP1 plays a functional role in hepatic tumor initiation, progression, or therapeutic response.

A disciplined target-assessment framework—emphasizing specificity, in-cell target engagement, therapeutic window definition, and systematic toxicity evaluation—will be critical before DCTPP1 can be credibly advanced toward clinical translation. Taken together, the available data position DCTPP1 as a promising but preliminary node within the complex metabolic landscape of cancer, underscoring the need for rigorous experimental validation to clarify its role in HCC.

## Figures and Tables

**Table 2:** Association between *DCTPP1* expression and overall survival across cancer types. Pan-cancer survival association of *DCTPP1* expression based on univariate Cox proportional hazards analysis using the TIMER3 platform. Data were obtained from the TCGA Pan-Cancer Atlas via TIMER3 (accessed on 15 July 2026). Z-score represents the standardized Cox regression coefficient (positive values indicate that higher *DCTPP1* expression is associated with increased mortality risk, whereas negative values indicate an association with improved survival). *p*-value indicates the univariate Cox regression *p*-value, and adjusted *p*-value represents the Benjamini–Hochberg false discovery rate (FDR)-adjusted *p*-value.

Cancers	z-Score	*p*-Value	Adjusted *p*-Value
ACC (*n* = 79)	1.12501849	0.260581199	0.445159549
BLCA (*n* = 406)	1.356209099	0.175032648	0.367167503
BRCA (*n* = 1086)	1.515902548	0.129543996	0.367167503
BRCA-Basal (*n* = 188)	−0.653593429	0.513373779	0.678978224
BRCA-Her2 (*n* = 82)	0.53960966	0.589466253	0.703967096
BRCA-LumA (*n* = 560)	1.452117965	0.146468807	0.367167503
BRCA-LumB (*n* = 216)	0.546587202	0.584662364	0.703967096
CESC (*n* = 304)	−0.458958549	0.646263933	0.703967096
CHOL (*n* = 35)	−0.173608426	0.86217321	0.88372754
COAD (*n* = 456)	−1.290601982	0.196841728	0.367847353
DLBC (*n* = 48)	−1.343512445	0.179106099	0.367167503
ESCA (*n* = 184)	−0.450351086	0.652457309	0.703967096
GBM (*n* = 287)	−0.904250057	0.365862823	0.555569472
HNSC (*n* = 520)	1.263656366	0.206353393	0.367847353
HNSC-HPV+ (*n* = 97)	0.234703299	0.814439027	0.856205131
HNSC-HPV− (*n* = 421)	1.48993084	0.136242422	0.367167503
KICH (*n* = 66)	2.539977523	0.0110859593	0.0505027036
KIRC (*n* = 533)	−4.634765038	3.57 × 10^6^	0.000146510683
KIRP (*n* = 290)	−1.025659814	0.305051963	0.48104348
LAML (*n* = 151)	2.85086566	0.00436003862	0.0255373691
LGG (*n* = 515)	2.876344841	0.0040230993	0.0255373691
LIHC (*n* = 371)	3.148776095	0.00163955764	0.0224072877
LUAD (*n* = 516)	1.085424516	0.277733679	0.455483234
LUSC (*n* = 501)	−1.365357244	0.172140795	0.367167503
MESO (*n* = 87)	0.497761547	0.618652122	0.703967096
OV (*n* = 422)	−0.68946671	0.490529616	0.670390475
PAAD (*n* = 178)	2.744918714	0.00605258875	0.0310195174
PCPG (*n* = 179)	0.52281058	0.601106077	0.703967096
PRAD (*n* = 497)	1.483412779	0.137964774	0.367167503
READ (*n* = 165)	−1.884017157	0.0595626547	0.244206884
SARC (*n* = 259)	2.954730549	0.00312942236	0.0255373691
SKCM (*n* = 470)	2.912733375	0.0035828037	0.0255373691
SKCM-Primary (*n* = 103)	0.618846659	0.536017381	0.68677227
STAD (*n* = 412)	−1.649192626	0.0991081782	0.367167503
TGCT (*n* = 134)	0.851853307	0.394295515	0.57736129
THCA (*n* = 505)	0.736191035	0.461614447	0.652627322
THYM (*n* = 120)	−1.267273706	0.205057439	0.367847353
UCEC (*n* = 545)	1.496948345	0.134406699	0.367167503
UCS (*n* = 57)	−0.125969696	0.899755913	0.899755913
UVM (*n* = 80)	−1.406069261	0.159703559	0.367167503

**Table 3:** Experimental and therapeutic strategies for targeting DCTPP1.

Models	Tools	Phenotypic Outcome	Reference
Breast cancer cells	Stable shRNA knockdown	Proliferation, colony formation, cell cycle	[7]
Gastric cancer cells	siRNA knockdown	5-fluorouracil (5-FU) sensitivity ↑	[21]
Ovarian cancer cells	shRNA knockdown	cisplatin sensitivity ↑, DNA damage ↑, apoptosis ↑	[24]
Various cancer cells	Small-molecule inhibitor Triptolide (DCTPP1 inhibitor)	DCTPP1 enzymatic activity ↓, proliferation ↓	[48]
Renal clear cell carcinoma cells	shRNA knockdown	proliferation ↓, invasion ↓	[15]

↑ indicates an increase, and ↓ indicates a decrease.

## Data Availability

No new experimental datasets were generated during this study. The structural model shown in Figure 1 was generated using PyMOL based on the publicly available crystal structure of human DCTPP1 (Protein Data Bank, PDB ID: 7MU5). Publicly available transcriptomic and survival datasets used for Figure 2 and Table 2 were obtained from the TCGA Pan-Cancer Atlas via the UCSC Xena Browser and the Tumor Immune Estimation Resource version 3 (TIMER3), respectively.
